# Intraperitoneal onlay mesh repair (IPOM) plus technique using a hybrid procedure of open laparotomy and laparoscopic approach (hybrid IPOM plus) for incarceration of umbilical hernia in a severely obese patient: a case report

**DOI:** 10.1186/s40792-020-00845-6

**Published:** 2020-04-26

**Authors:** Shingo Yanari, Takayuki Suto, Hisataka Fujiwara, Yu Ariyoshi, Akira Umemura, Akira Sasaki

**Affiliations:** 1 Department of Surgery, Morioka Municipal Hospital, 5-15-1 Motomiya, Morioka, Iwate, 020-0866 Japan; 2 Department of Surgery, Iwate Medical University School of Medicine, 1-1-1 Idaidori, Yahaba-cho, Shiwa-gun, Iwate, 028-3694 Japan

**Keywords:** Severely obese patient, Incarceration of umbilical hernia, Hybrid intraperitoneal onlay mesh repair (IPOM) plus

## Abstract

**Background:**

A standard procedure for the treatment of incarcerated umbilical hernia among severely obese patients has yet to be established. We used the hybrid intraperitoneal onlay mesh repair (IPOM) plus method, which combines open and laparoscopic surgery to treat incarcerated umbilical hernia in a severely obese patient.

**Case presentation:**

A 46-year-old man presented in our department with a chief complaint of a painful mass in the umbilical region. Incarcerated umbilical hernia was diagnosed on the basis of abdominal computed tomography, and the decision was made to perform emergency surgery. The patient was severely obese (body mass index, 53.8 kg/m^2^), and the incarcerated portion of the hernia was therefore first addressed by open surgery. As bowel resection was unnecessary, the risk of infection was considered low, and after direct closure of the hernia orifice, IPOM was performed laparoscopically using the hybrid IPOM plus method.

**Conclusion:**

Among severely obese patients, first trocar insertion is difficult and the wound site tends to come under strain, meaning that simple closure of the hernia orifice results in a high recurrence rate. The hybrid IPOM plus method used in this case combines open surgery and laparoscopy and appears useful for treating uninfected incarcerated umbilical hernia in severely obese patients safely and with an anticipated low rate of postoperative recurrence.

## Background

Adult umbilical hernias are believed to occur due to acquired weakness in the umbilical ring after closure [1]. Causes include retention or production of a large volume of ascites due to cirrhosis of the liver or similar disorders, as well as increased abdominal pressure due to causes such as severe obesity. In severely obese patients with body mass index (BMI) 30 kg/m^2^, umbilical hernias show a postoperative recurrence rate of 31.8%, far greater than the 8.1% in patients with BMI < 30 kg/m^2^ [2].

In the intraperitoneal onlay mesh repair (IPOM) method of abdominal wall hernia repair, mesh is placed over the abdominal wall defect and secured from inside the peritoneal cavity [3]. In the IPOM plus method, in addition to IPOM, the hernia orifice is also closed by direct suturing, a procedure that is anticipated to reduce the recurrence rate [4–6]. Laparoscopic surgery in severely obese patients, however, is a difficult procedure, as subcutaneous fat and visceral fat make the first trocar insertion difficult, in addition to other problems, including restricted port mobility [7, 8].

We report herein our use of the hybrid IPOM plus method combining open and laparoscopic surgery to safely treat incarcerated umbilical hernia in a severely obese patient with a BMI of 53.8 kg/m^2^, together with a discussion of the literature.

## Case presentation

A 46-year-old man with a history of diabetes, fatty liver, hypertension, and dyslipidemia had been aware of periumbilical pain since the previous day. In addition to this pain, he had also developed other abdominal symptoms including abdominal discomfort and vomiting. He was admitted to our hospital complaining of painful mass in the umbilical region for examination.

**Fig. 1:**
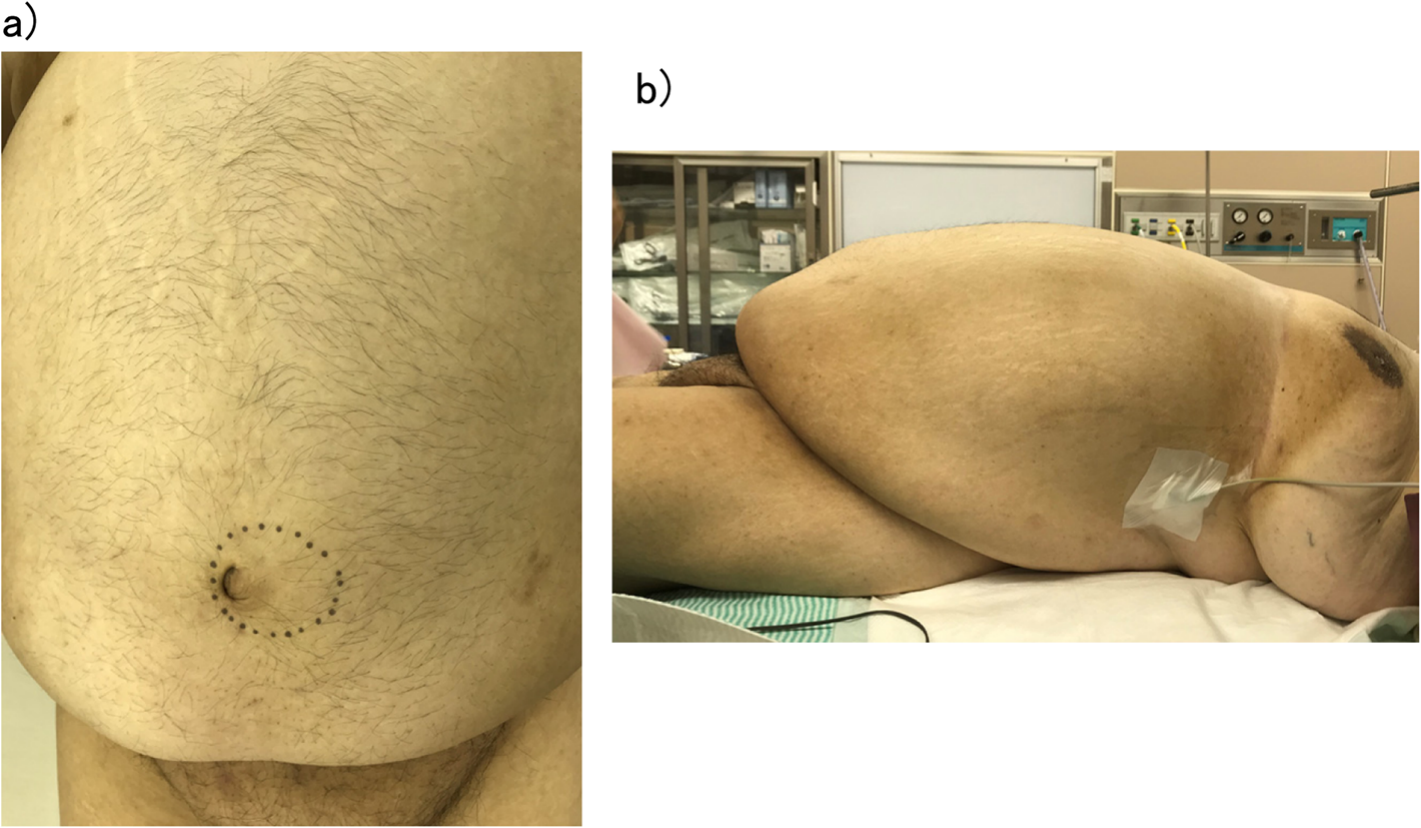
Abdominal photographs. **a** Frontal view: a mass measuring approximately 10 cm is present in the umbilical region (dotted circle). **b** Side view

His examination on presentation was height, 165 cm; weight, 146.6 kg; BMI, 53.8 kg/m^2^; temperature, 36.8C; blood pressure, 142/88 mmHg; heart rate, 86 beats/min; periumbilical pain; and umbilical mass (Fig. 1a, b).

His blood test results on presentation included signs of mild inflammation, with a white blood cell count of 6500/mm^3^ and a C-reactive protein (CRP) concentration of 3.09 mg/dL, and signs of mild dehydration with blood urea nitrogen (BUN) of 25.1 mg/dL and creatinine of 1.08 mg/dL.

**Fig. 2:**
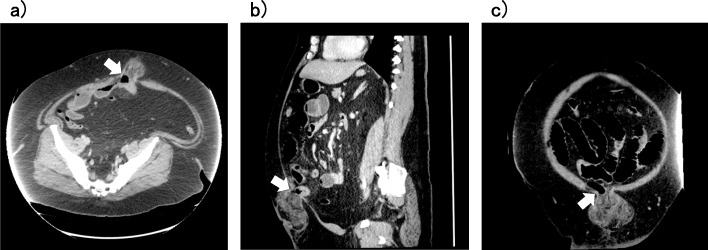
Abdominal contrast-enhanced CT. The greater omentum and small intestine appear incarcerated (arrow). **a** Horizontal section. **b** Coronal section. **c** Sagittal section

Abdominal contrast-enhanced computed tomography (CT) showed high-density subcutaneous fat and hernia in the umbilical region, with the hernia containing greater omentum and small intestine. Incarcerated umbilical hernia was therefore diagnosed (Fig. 2a–c). No dilation of the gastrointestinal tract was apparent.

On the basis of these findings, the hernia contents were considered to mainly comprise greater omentum and that this was therefore a case of incarcerated Richter umbilical hernia. The decision was made to perform emergency surgery. The patient was severely obese (BMI, 53.8 kg/m^2^), and as difficulties with first trocar insertion and securing sufficient working space for laparoscopic operations were anticipated, we decided to first release the incarcerated umbilical hernia by open surgery. If bowel resection is required, the hernia orifice is closed by simple suturing alone, whereas when bowel resection is unnecessary, the IPOM plus method using a mesh is chosen to reduce the risk of recurrence. In this case, we therefore adopted a strategy of using a hybrid IPOM plus method combining open and laparoscopic surgery.

**Fig. 3:**
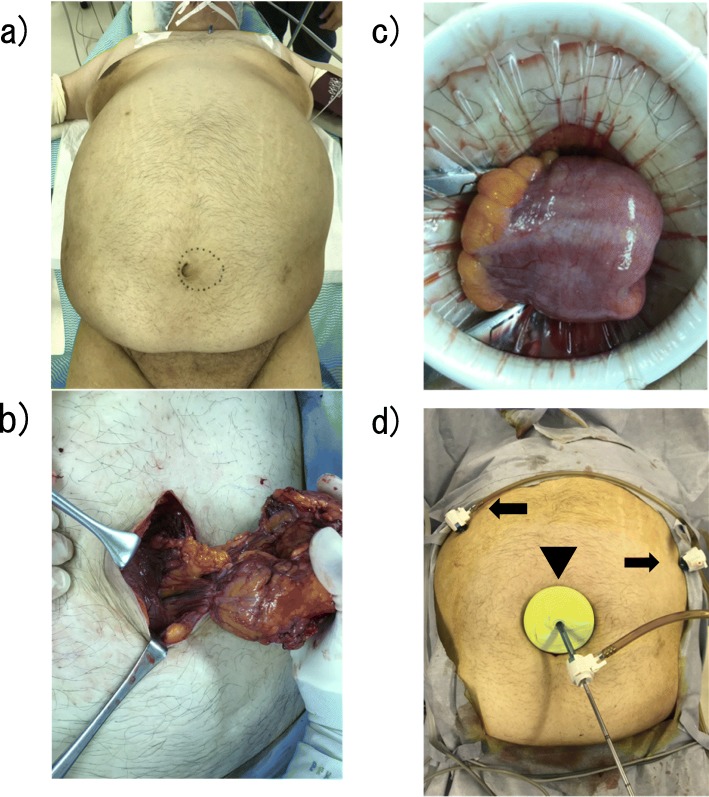
Incarcerated umbilical hernia release. **a** Surgical position. **b** Incarceration of the omentum in the hernia sac is evident. **c** No perforation or necrotic changes are evident in the incarcerated small intestine. **d** Alexis® laparoscopic system (arrowhead) and 5-mm trocar (arrow)

**Fig. 4:**
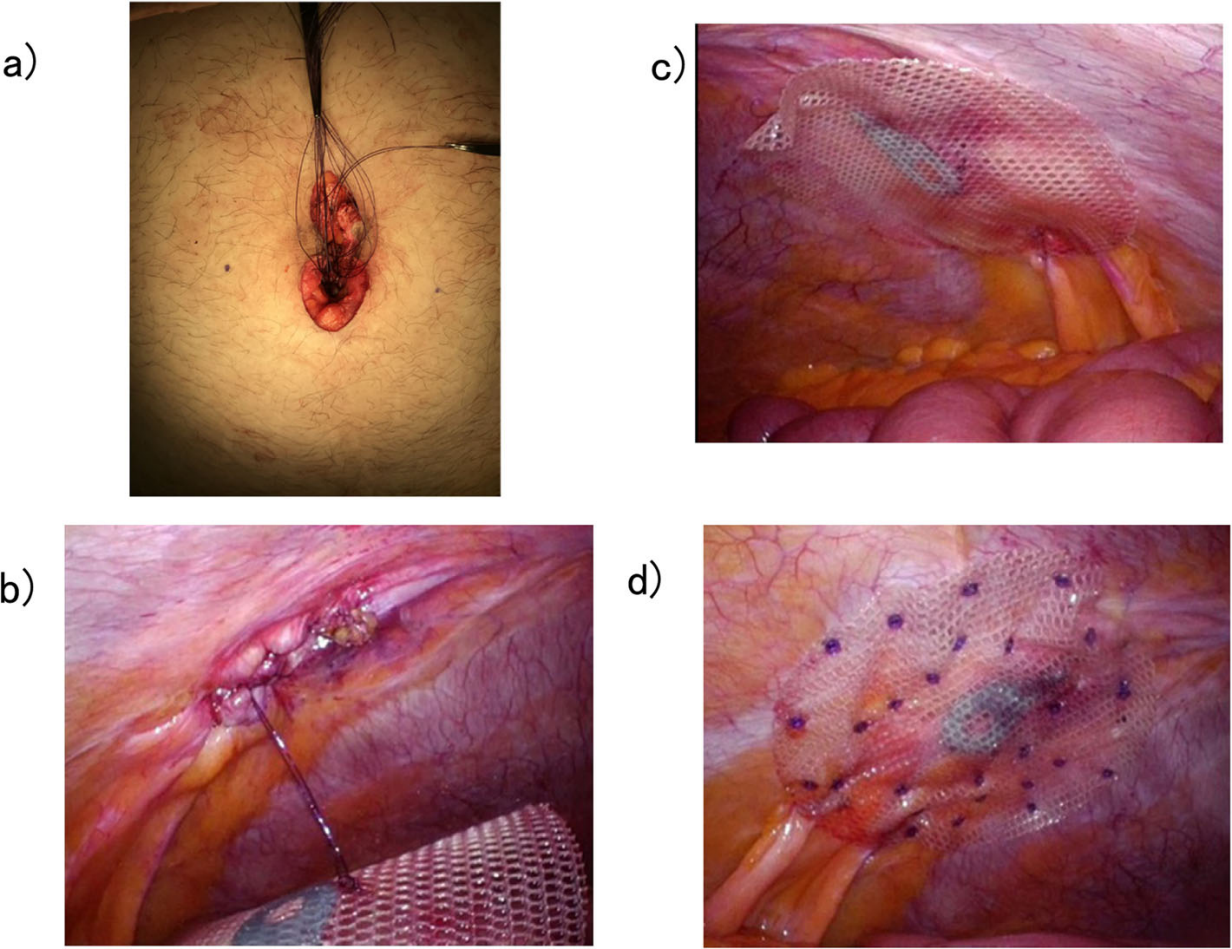
Curative surgery for umbilical hernia. **a** Closure of the hernia orifice with simple interrupted sutures. **b** The hernia orifice after suture closure observed from inside the peritoneal cavity. **c** Temporary securing of the mesh. **d** Securing the mesh using the double-crown method

**Fig. 5:**
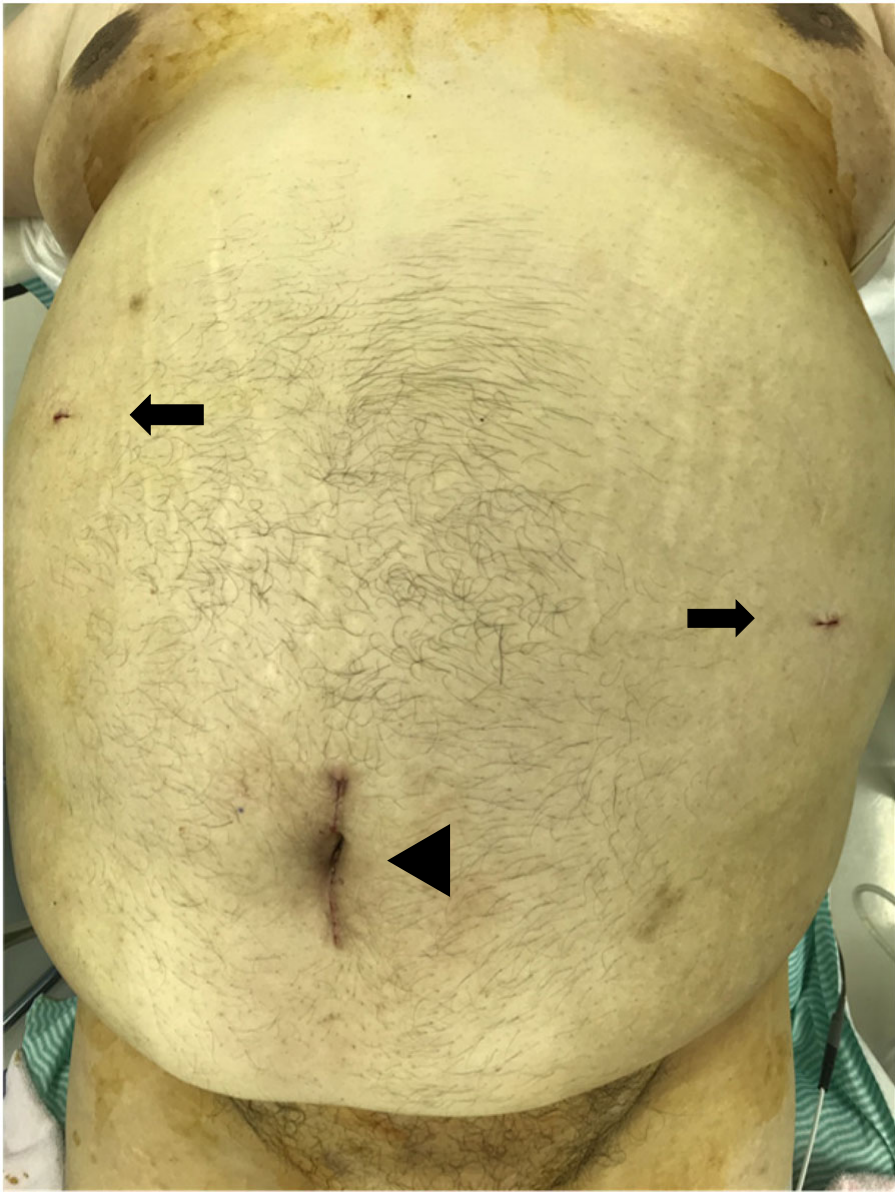
Skin incision wound immediately postoperatively. The Alexis® laparoscopic system insertion site (arrowhead) and insertion sites for 5-mm trocars (arrows) are evident

Surgery was performed under general anesthesia by tracheal intubation without concurrent use of epidural anesthesia (Fig. 3a). A 10-cm longitudinal skin incision with the umbilicus at the center was created to expose the hernia sac, which was mobilized. The hernia contents comprised greater omentum and small intestine. The incarcerated greater omentum could not be reduced to the peritoneal cavity and was resected (Fig. 3b). No perforations or necrotic changes to the incarcerated small intestine were identified, and bowel resection was judged unnecessary. Reduction to the peritoneal cavity was therefore performed (Fig. 3c). After hernia sac resection, the long diameter of the hernia orifice was measured (6 cm). The mesh used was a 12-cm Symbotex™ round mesh (Covidien, Mansfield, MA, USA) coated with collagen to prevent adhesions and was sufficiently large to overlap the hernia orifice by at least 3 cm. Using a Alexis® laparoscopic system S size (Applied Medical, Rancho Santa Margarita, CA, USA), a 5-mm ENDOEYE FLEX™ deflectable-tip videoscope (Olympus, Tokyo, Japan) was inserted via this site under insufflation pressure of 10 mmHg, and 5-mm trocars were inserted at two sites, under the right costal arch and on the cranial side of the left umbilical region (Fig. 3d). The insufflation was released, and the mesh was inserted into the peritoneal cavity. The hernia orifice was closed by simple interrupted sutures with 0 VICRYL™ (Ethicon, Cincinnati, OH, USA) (Fig. 4a, b), insufflation was again performed, and supporting sutures were placed on the cranial and caudal sides of the mesh using an EndoClose™ (Covidien) to temporarily secure the orifice by pulling it up against the abdominal wall (Fig. 4c). This mesh was further secured by the double-crown method using an AbsorbaTack™ (Covidien) (Fig. 4d). The umbilical wound was closed by dermal suturing with 4-0 Biosyn™ (Covidien) (Fig. 5). Lidocaine hydrochloride (1%) was infiltrated into trocar sites and small open wound at the end of surgery in addition to diclofenac sodium suppository (50 mg) administration. The operating time was 100 min, and intraoperative hemorrhage was 160 ml. The patient took celecoxib 400 mg/day orally for 4 days from the day of surgery.

Postoperative course was uneventful, and no additional analgesics were needed. The patient was discharged on day 5. He underwent dietary therapy as an outpatient, and at 6 months postoperatively, his BMI had decreased to 43.2 kg/m^2^, with no recurrences identified as of the time of writing.

## Discussion

The first umbilical hernia repair procedure was described by William Cheselden in 1740 [9]. Since 1901, when William J. Mayo reported a method of suturing by overlapping fascia using nonabsorbable sutures [10], either simple closure or the Mayo technique has been used, but the recurrence rate remains at 10–30% [11]. In a randomized clinical trial comparing simple closure with mesh use in umbilical hernia surgery, no difference was evident in early complications, but the recurrence rate was significantly lower when mesh was used [11]. In patients with large hernia orifices, the IPOM method not only shows a high recurrence rate but also is associated with problems such as seroma and mesh bulge, and the IPOM plus method is therefore recommended [4–6]. The IPOM plus method is currently regarded as the best surgical procedure for umbilical hernia repair.

The IPOM and IPOM plus methods can both be performed either by open surgery or laparoscopically, but the incisional wound is much larger in purely open surgery. Among severely obese patients, laparoscopic surgery is minimally invasive but difficult, as subcutaneous and visceral fat make insertion of the first trocar difficult in addition to other problems, including restricted port mobility [7, 8]. The small-incision technique and optical technique can be used in laparoscopic approaches for severely obese patients. The optical technique requires a special trocar and expertise in its use. In this case, we used the small-incision technique. Although the incision required to release the incarcerated hernia was smaller than that used for open surgery, it was still larger than usual in the small-incision technique, at 10 cm, providing a good field of view. Using the small incision for omental resection and to check for ischemia of the small intestine, we were able to complete these procedures easily and within a short time. In cases of non-incarcerated umbilical hernia, preoperative weight loss followed by elective surgery should be considered. However, a standard procedure for emergency surgery in cases of incarcerated umbilical hernia in severely obese patients has yet to be established, and we considered that hybrid surgery utilizing the respective advantages of both open and laparoscopic surgery provides a useful technique in emergency surgery for severely obese patients as in the present case.

If resection of the incarcerated bowel is required, the risk of infectious ascites due to intestinal necrosis or contamination with intestinal bacteria as a result of bowel resection is high, and mesh use should be avoided in such cases. Mesh use is contraindicated in the event of contaminated surgery, including abscess formation, intestinal perforation, serious peritonitis, and the presence of severe infection in the operating field itself [12]. The safety of mesh use in clean-contaminated surgery such as small bowel resection remains unclear [13]. We do not use mesh if contamination is suspected, such as if combined intestinal resection has been performed. In this case, we decided to first perform open surgery, then to choose between simple closure and IPOM plus, depending on the risk of infection after release of the incarcerated hernia. The hybrid IPOM plus method is a procedure that can be flexibly used in either event.

Port-site hernia is a complication specific to laparoscopic surgery, and obesity is one risk factor [14]. A port site measuring 10 mm is reportedly the cause of 86% of cases of port-site hernia [15]. To prevent port-site hernia in the present case, we decided not to use 10-mm trocars and were able to complete the operation easily using only two 5-mm ports for the laparoscope and forceps. This was because following the open surgery, we were able to use a laparoscopic system to observe the insufflated peritoneal cavity and simulate the optimum insertion sites for the 5-mm trocars. However, although it is important in surgery on severely obese patients to start with the least number of trocars of smallest size, if such a strategy would render the surgery infeasible, surgeons should not hesitate to consider increasing the number or changing to a larger size.

## Conclusion

We treated a severely obese patient with incarcerated umbilical hernia. In severely obese patients, first trocar insertion is difficult, the wound tends to come under strain, and the recurrence rate after simple closure alone is high. The hybrid IPOM plus method used to treat this case of incarcerated umbilical hernia in a severely obese patient enabled both the first trocar to be inserted safely and a flexible choice of procedure in light of whether infection was present. We consider that surgery completed by this method is safe and effective and likely to reduce the recurrence rate.

## Data Availability

Not applicable

## Abbreviations

BMI: Body mass index
BUN: Blood urea nitrogen
CRP: C-reactive protein
CT: Computed tomography
IPOM: Intraperitoneal onlay mesh repair
